# Glycated Albumin and Angiopoietin-2: Possible indicators of Diabetic Retinopathy in Type-Two Diabetes

**DOI:** 10.12669/pjms.38.8.5579

**Published:** 2022

**Authors:** Kanwal Ijaz, Muhammad Luqman Ali Bahoo, Sobia Niaz, Hafiz Usman Ahmad

**Affiliations:** 1 Dr. Kanwal Ijaz, MPhil. Assistant Professor, Department of Physiology, Azra Naheed Medical and Dental College, Lahore, Pakistan; 2 Dr. Muhammad Luqman Ali Bahoo, FCPS, FICO, FACS. Fellowship in Refractive & Corneal Surgery Professor and Head, Department of Ophthalmology, CMH Institute of Medical Sciences, Bahawalpur, Pakistan; 3 Dr. Sobia Niaz, DCH, MPhil. Associate Professor, Department of Physiology, Gujranwala Medical College, Gujranwala, Pakistan; 4 Hafiz Usman Ahmad, MLT. Department of Physiology and Cell Biology, University of Health Sciences, Lahore, Pakistan

**Keywords:** Diabetes mellitus, Diabetic retinopathy, Markers, Morbidity, Mortality

## Abstract

**Objectives:**

To compare the levels of glycated albumin and angiopoietin-2 in Type-Two diabetics with and without diabetic retinopathy.

**Methods:**

It was a cross-sectional comparative study done at University of Health Sciences, Lahore after collecting data from recruited patients from the outpatient department of Layton Rahmatulla Benevolent Trust Eye Hospital, Lahore from 1^st^ July, 2016 to 30^th^ Aug., 2017. A total of 80 type two diabetics of both genders fulfilling the inclusion criteria were included and divided in two groups based on absence and presence of diabetic retinopathy. Obtained data were analyzed using IBM SPSS for Windows software (version 22). For comparison of both groups, Independent “t” Test or Mann-Whitney U tests were applied accordingly. For correlation of quantitative variables in each group, Spearman rho correlation and Pearson correlation test were applied depending upon normality of data.

**Results:**

Among 80 type-two diabetics, 42 (52.5%) patients had diabetic retinopathy and 38 (47.5%) were without diabetic retinopathy. Overall, females (62.5%) outnumbered males (37.5%). Both study group were age matched (*p*=0.45). Mean serum albumin in diabetic retinopathy and non-diabetic retinopathy group was 4.20 ±0.56 gm/dL and 4.43 ±0.39 gm/dL respectively (*p*=0.031). In diabetic retinopathy group, mean glycated albumin was 1.48 (0.63-1.76) gm/dL and median IQR in non-diabetic retinopathy was 0.52 (0.23-1.10) gm/dL (*p*=0.003). In diabetic retinopathy group, mean glycated albumin (percent) was 30.71±18.63% and in non-diabetic retinopathy group, the median IQR was 11.80 (5.06-27.25) (*p*= 0.001). The angiopoietin-2 median IQR in diabetic retinopathy group 5.70 (5.47-5.80) was significantly different (*p=*0.033) from diabetics without diabetic retinopathy groups 5.40 (4.97-5.60).

**Conclusion:**

Our study reported **r**aised levels of glycated albumin (percent) and angiopoietin-2 in type-two diabetics, highlighting their possible involvement in disease and its progression.

## INTRODUCTION

Diabetes mellitus is among the very commonly prevailing illnesses in Pakistan. The existing prevalence of type-2 diabetes mellitus in Pakistan is 11.77%.^1^ It makes the Pakistan to be on the position seven in the world with large number of diabetic patients. Furthermore, it may become 4^th^ largest by the year 2030 as stated by the report.^2^

Marked hyperglycemia in diabetes mellitus (DM) is responsible for morbidity and mortality owing to its complications which either effect microvasculature such as in retina (retinopathy), in nerves (neuropathy) and in kidney (nephropathy) or effect macrovasculature leading to cerebrovascular accident, coronary heart disease and peripheral vascular disease.^3^ Sixty percent of diabetic patients develop diabetic retinopathy within 20 years of disease and 33% of blindness in older age is due to diabetic retinopathy.^4^ Microvascular injury occurs to innermost lining of retina in eyes which leads towards the loss of vision.^5^ Visual loss from diabetic retinopathy (DR) is both avoidable and curable.^6^ Duration and type of diabetes, blood glucose levels, lipid profile and elevated blood pressure are the basic elements of progression and development of DR.^5^ Moreover, once DR develops in diabetic patients it significantly predisposes the patient to develop other macrovascular as well as microvascular problems.^7^ Retinopathy of diabetes is classified as non-proliferative diabetic retinopathy (NPDR) and proliferative diabetic retinopathy (PDR). Non-proliferative diabetic retinopathy have mild, moderate and severe grades; considered by presence of micro aneurysm, retinal hemorrhages and soft or hard exudates. Whereas, proliferative diabetic retinopathy (PDR) which involves development of new vessels (neovascularization) with or without vitreal hemorrhage on manual examination of fundus with the help of ophthalmoscope. Diabetic macular edema may or may not be present in both.^5^

The precise mechanism through which various diabetic risk factors initiate the vascular damage is still unclear, however, various interconnecting pathways involved in pathogenesis of DR are proposed. Mainly, it includes increased polyol pathway flux, diacylglycerol–PKC pathway activation, advance glycated end products formation and raised hexosamine pathway flux. All these conditions lead towards increased production of growth and angiogenic factors; inflammation; leukostasis; initiation of renin-angiotensin-aldosterone system (RAAS) and oxidative stress.^8^

Glycated albumin (GA) is an amadori ketoamine developed by the non-enzymatic glycation of serum albumin in diabetics.^9^ Glycated albumin, an intermediate of advanced glycated end products, represent almost 80% of the total of glycations in plasma.^10^ Glycated albumin levels are increased in the presence of hyperglycemia. It reflects glycemic control over the last three weeks. This property of GA is utilized as a marker of control of glucose levels in blood.^9^ In type two diabetics, GA is significantly correlated with HbA1c and fasting glucose levels. Though measuring HbA1c is gold standard for monitoring mean glycemia over the last 2-3 months, but in conditions where HbA1c test may be unreliable, or earlier clinical decision making is mandatory, GA can be useful complimentary biomarker for measuring blood glucose fluctuation over last three weeks.^11^ Raised GA levels have a significant correlation with the presence of diabetic retinopathy.^12^ High levels of advance glycated end products (AGEs) in diabetics stimulates endothelial cells and cause up-regulation of mRNA of angiopoietin-2 (ANG-2).^13^ Subsequently ANG-2 causes endothelial damage and abnormal angiogenesis.^14^ Hyperglycemia alters the levels of vascular endothelial growth factor (VEGF), angiopoietin one and two and recruits activated macrophages which lead to loss of capillary pericytes and subsequent development of non-perfused capillaries. Loss of microvascular integrity is the crucial step in response to hyperglycemia in diabetes.^15^

Despite the fact that pathogenic basis of DR is not completely understood at cellular and molecular level, efforts are going on worldwide to explore the new and effective mediators in reference to early diagnosis and treatment of DR.

Currently in clinical practice, to establish the diagnosis of DR & its risk factor, Common biomarkers include visualization of the retinal vasculature and to measures blood glucose, lipids, blood pressure, body weight and smoking. Greater knowledge of novel biomarkers and mediators of diabetic retinopathy, such as those related to inflammation and angiogenesis, has contributed to the development of additional therapeutics.^16^

Almost all the treatment options are targeted to treat damaged retina at an advance stage of DR. However, efforts should be made to identify biomarkers for early diagnosis and subsequently effective treatment options at asymptomatic stage to prevent the loss of vision.

The current study compared and reported the serum GA and ANG-2 levels in patients of type-two diabetes mellitus (T2DM) with and without diabetic retinopathy in the local population. This study may provide some insight into levels of GA and ANG-2 in patients of DR and subsequently their possible implication for early detection of retinopathy in our setting.

## METHODS

This cross-sectional comparative study was conducted from 1^st^ July, 2016 to 30^th^ August, 2017 after approval of the Ethical Review Committee of University of Health Sciences, Lahore (UHS/Education/126-16/1166).

It included 80 type two diabetic patients from the outpatient department of Layton Rahmatulla Benevolent Trust Eye Hospital, Lahore. Detailed assessment of previous medical history was done to exclude type two diabetics with sepsis, tumor, overt kidney disease, liver or thyroid disease. Written informed consent was taken. Each subject was assessed by performing physical examination, using specially designed Proforma.

Diabetic retinopathy (DR) status was accessed by an expert consultant ophthalmologist through indirect ophthalmoscopy with the help of Superfield 90D lens. Subjects were categorized in two groups based on the presence and absence of diabetic retinopathy. Staging of DR was done according to the standards.^17^ Venous blood samples were taken using standard techniques with the help of sterilized needles.

Biochemical analysis was done in the Department of Physiology and Cell biology of University of Health Sciences, Lahore. Serum was separated from blood by centrifuging the blood at a speed of 3000 revolutions per minute (rpm) for ten minutes, within two hours after sample collection. Serum albumin and glycated albumin were estimated by spectrophotometry while angiopoietin-2 was determined by enzyme linked immunosorbent assay (ELISA). Glycated albumin can be described as an absolute concentration (mg/ml) or as a percent (%) of total albumin. It can be calculated from the following equation:

% Glycated Albumin _sample_ = 100% x Glycated albumin _sample_ / Total Albumin _sample_^18^

From the levels of serum albumin and glycated albumin, glycated albumin percent was calculated. Obtained data were analyzed using IBM SPSS for Windows software (version 22). For comparison of both groups, Independent “t” Test or Mann-Whitney U tests were applied accordingly. For correlation of quantitative variables in each group, Spearman rho correlation and Pearson correlation test were applied depending upon normality of data.

## RESULTS

In current study, 80 patients with type two diabetes mellitus (T2DM) were included which were further divided on the basis of presence of diabetic retinopathy into two groups i.e., diabetic retinopathy group (42) and diabetic without retinopathy group (38). Females outnumbered males in both groups (Table-I).

**Table-I:** Descriptive analysis of qualitative variables.

Qualitative variables	Categories	T2DM with diabetic retinopathy n=42(%)	T2DM without diabetic retinopathy n=38(%)
Gender	Males	17(40.48)	13(34.20)
	Females	25(59.52)	25(65.8)
Eye involvement	Unilateral	5(11.90)	0(0.00)
	Bilateral	37(88.10)	0(0.00)

**Fig.1:**
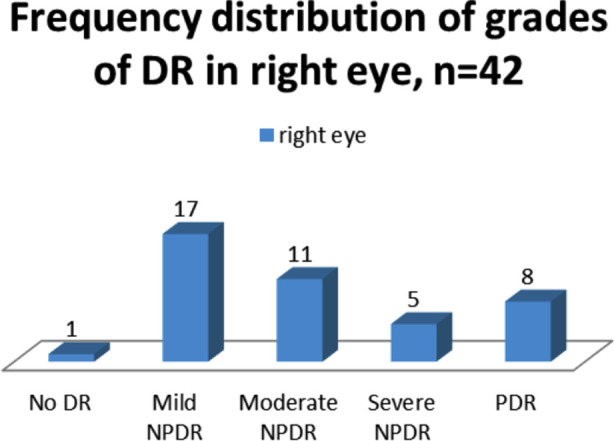
Frequency distribution of grades of diabetic retinopathy in right eye.

**Fig.2:**
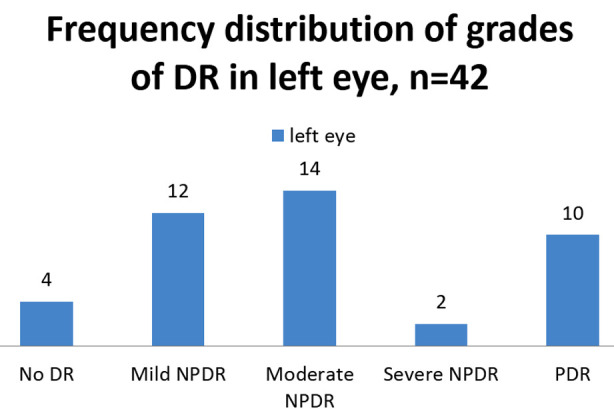
Frequency distribution of grades of diabetic retinopathy in left eye.

Diabetic retinopathy group had varying grades of diabetic retinopathy in both eyes (Fig. 1 & 2). Median (IQR) age in diabetic retinopathy group was 53.00 (49.75-60.00) and in diabetic without retinopathy group 55.00(50.00-58.25) years. A non-significant difference was analyzed on comparison of ages of two groups by Mann Whitney U test (*p*=0.45, Table-II).

**Table-II:** Comparison of study variables in Type-2 diabetics (T2DM) with and without diabetic retinopathy (DR).

Parameters	Type-2 diabetics with diabetic retinopathy n=42	Distribution	Type-2 diabetics without diabetic retinopathy n=38	Distribution	p-value
	
	Mean ± SD/ Median(IQR)		Mean ± SD/ Median(IQR)		
Age (years)	53.00(49.75-60.00)	Non-normal	55.00(50.00-58.25)	Non-normal	0.453^b^
Duration of diabetes (years)	8.50(5.00-12.00)	Non-normal	7.50 (5.00-10.00)	Non-normal	0.042*^b^
Total albumin (gm/dL)	4.20 ±0.56	Normal	4.43 ±0.39	Normal	0.031*^a^
Glycated albumin (gm/dL)	1.26 ±0.76	Normal	0.52 (0.23-1.10)	Non-normal	0.003*^b^
Glycated albumin percent (%) (of total albumin)	30.71 ±18.63	Normal	11.80 (5.06-27.25)	Non-normal	0.001*^b^
Angiopoietin-2 (ng/mL)	5.70 (5.47-5.80)	Non-normal	5.40 (4.97-5.60)	Non-normal	0.033*^b^

a p value is generated by student t-test

b p value is generated by Mann Whitney U test

* p-value ≤ 0.05 is considered statistically significant.

Median (IQR) diabetes duration (years) in diabetic retinopathy group, was 8.50 (5.00-12.00) while in diabetic non-retinopathy group, it was 7.50 (5. 00-10.00). It was observed by Mann Whitney U test that there was significant difference between Median (IQR) of duration of diabetes of both the groups (*p*=0.042, Table-II).

Diabetic retinopathy group had mean±SD 4.20 ±0.56 gm/dL while diabetic non-retinopathy group had mean±SD 4.43 ±0.39 gm/dL of albumin and significant difference was observed by Student “t” test (*p*=0.031; Table-II).

In diabetic retinopathy group, mean±SD glycated albumin (gm/dl) was 1.26 ±0.76 and median IQR in diabetic non-retinopathy was 0.52 (0.23-1.10) respectively. A significant difference was observed by Mann Whitney U test (*p*=0.003, Table-II).

In diabetic retinopathy group, mean±SD percent glycated albumin (percent) was 30.71 ±18.63% and in diabetic non-retinopathy group, the median IQR was 11.80 (5.06-27.25). A significant difference was observed by Mann Whitney U test (*p*= 0.001; Table-II).

The median IQR of angiopoietin-2 in diabetic retinopathy group and in diabetics without diabetic retinopathy groups was 5.70 (5.47-5.80) ng/ml and 5.40 (4.97-5.60) respectively (*p=*0.033, Table-II). Angiopoietin-2 showed positive significant correlation with albumin (*p*=0.008) and glycated albumin (*p*=0.005) in diabetic retinopathy group (Table-III).

**Table-III:** Correlation of biochemical variables with angiopoietin two in type-2 diabetics.

Angiopoietin-2 (ng/mL)				

Parameters	Type-2 diabetics with diabetic retinopathy N=42		Type-2 diabetics without diabetic retinopathy N=38	

	r / rho	p-value	r / rho	p-value
Total albumin (gm/dL)	0.406^b^	0.008*	0.241^b^	0.145
Glycated albumin(gm/dL)	0.423^b^	0.005*	0.041^b^	0.805
Glycated albumin percent (%) (of total albumin)	0.302^b^	0.052	0.035^b^	0.834

^a^ Correlation coefficient (r) and p-values are generated by Pearson Correlation Coefficient

b Correlation coefficient (rho) and p-values are generated by Spearman’s Rho Correlation Coefficient

* p -value ≤ 0.05 is considered statistically significant.

## DISCUSSION

In present study we compared the levels of, glycated albumin and angiopoietin-2 between type two diabetics with and without diabetic retinopathy. Our study reported the high serum levels of glycated albumin, glycated albumin (per cent) and angiopoietin two in type two diabetics with diabetic retinopathy as compared to non-retinopathy diabetics with significant differences (*p=*0.003, *p=*0.001 and (*p*=0.033) respectively, for GA, GA (per cent) and ANG-2).

Diabetes complications are directly related to duration of diabetes and poor glycemic control.^5^ Uncontrolled hyperglycemia of DM results in non-enzymatic alterations of many plasma proteins e.g. glycation of serum albumin (which represents about 80% of the total of glycation in plasma)^10^ causes very harmful effects both in development as well as in progression of diabetic complications.^19^ Advance glycation end products (AGEs) dominate in the vasculature of patients of DM^20^ and are believed to play an important role in the pathogenesis of diabetic retinopathy.^21^

For the screening of diabetes and its associated risk factors, measurement of GA is a useful constituent of routine physical examinations.^22^ Jeon *et al*. also reported the significantly higher levels of GA and GA% in subjects with T2DM having diabetic retinopathy as compared to T2DM patients with no retinopathy. He also reported significantly higher levels of glycated albumin (percent) in diabetic patients with retinopathy than those without DR.^23^ Our results are similar to a recent study of which explored the association between the incidence of DR and the GA/HbA1c and reported the association of DR with GA/HbA1c ratio.^24^ The presence of diabetic retinopathy is significantly correlated with increased GA levels.^9^ Measuring GA levels in addition to HbA1c was beneficial as a marker for retinopathy, especially in patients with moderate glycemic control.^12^ Glycated albumin may be a more valuable glycation index than HbA1c for checking glycemic control in type-2 diabetics who has very fluctuating and badly controlled glycemic conditions.^25^ In our study, the higher levels of GA in diabetic retinopathy group validates the fact that poorly controlled blood sugar level reflected itself as higher GA levels which most probably facilitated in development of diabetic retinopathy.

The study of Torimoto *et al*. supports the fact that variations in levels of blood glucose play a substantial role in dysfunction of vascular endothelial layer and adds to development of angiopathies in type-2 diabetes.^26^ Both groups, in the present study, had statistically significant different levels of angiopoietin-2 (*p*=0.033). Similar to our results, Li *et al*. also reported that diabetic patients have higher levels of ANG-2.^14^ Earlier, Patel *et al*. measured the ANG-2 levels in vitreous of eyes of patients with DR and reported higher ANG-2 levels in NPDR.^27^ Lip *et al*. reported that the VEGF (*p*=0.001) and ANG-2 levels (*p*=0.001) were significantly higher in 93 type-2 diabetic patients as compared to 20 healthy ones with the highest levels in grade-2 and grade-3 diabetic retinopathy (*p*=0.05).^28^ Loss of microvasculature integrity due to damage of pericytes is the vital step in the development of DR and ANG-2 has significant role in this pathology.^15^ When correlation of angiopoietin-2 with other parameters was measured, there was a positive correlation with serum albumin and glycated albumin (*p*=0.008 and *p*=0.005; respectively). Our study supported the earlier known fact that higher levels of AGEs in diabetics stimulates endothelial cells and cause up-regulation of mRNA of angiopoietin-2.^13^ Subsequently, ANG-2 causes endothelial damage and abnormal angiogenesis^14^ which is a hallmark of DR.

### Limitations:

It is a cross sectional study so a casual association cannot be denoted further in a single center study. The main limitation of our study was the limited number of samples and lack of assessment of HbA1c to explore the correlation between them, due to financial constraints.

## CONCLUSIONS

This study reports that diabetic patients with diabetic retinopathy have higher levels of glycated albumin and angiopoitin-2 as compared to non-DR diabetics. The significant positive correlation of glycated albumin and angiopoietin-2 in individuals with diabetic retinopathy suggests an interrelated pathway in the genesis and progression of the disease.

### Recommendations:

Special attention should be paid to achieve normal blood glucose levels in order to prevent the development of advanced glycation end products. Further studies, with higher number of patients, should be conducted to highlight the relationship of other molecules involved in the pathogenesis of different micropathies as well.

### Authors’ Contribution:

**KI:** Data collection, data analysis and manuscript writing. Accountable for the accuracy or integrity of the work.

**MLAB:** Conceived, designed and reviewed the manuscript.

**SN:** Helped in drafting and final reviewing the manuscript.

**HUA:** Helped in lab analysis and data compilation.
